# Assessing Teaching Effectiveness in Blended Learning Methodologies: Validity and Reliability of an Instrument with Behavioral Anchored Rating Scales

**DOI:** 10.3390/bs12100394

**Published:** 2022-10-16

**Authors:** Luis Matosas-López, Elena Cuevas-Molano

**Affiliations:** 1 Department of Financial Economics and Accounting, Rey Juan Carlos University, P.º de los Artilleros, s/n, 28032 Madrid, Spain; 2 Department of Communication Sciences and Sociology, Rey Juan Carlos University, C/ Camino del Molino, 5, 28942 Madrid, Spain

**Keywords:** teaching effectiveness, blended learning, validity, reliability, behavioral episodes, behavioral examples, behavioral scales, BARS

## Abstract

The evaluation of teaching effectiveness in blended learning methodologies is usually carried out using Likert-type questionnaires; however, instruments with Behavioral Anchored Rating Scales (BARS) are sometimes employed for this purpose. This paper examines the validity and reliability of an instrument with BARS designed to assess teaching effectiveness in blended learning environments, within the university setting. The research involves a sample of 1436 students from a medium size university in Spain. Using this sample (n = 1436), the authors carry out a psychometric study that consists of four phases: (1) comprehension validity analysis, (2) construct validity analysis, (3) confirmation of construct validity, and (4) analysis of the instrument reliability. The findings provide satisfactory values for all the parameters analyzed (for instance: Variance explained = 77.61%; RMSEA = 0.042; or Cronbach’s alpha = 0.956), indicating that the BARS instrument examined is perfectly valid and reliable for the appraisal of teaching effectiveness in blended learning methodologies. The authors conclude that this paper fills an important gap in the literature by presenting an instrument that, thanks to the use of behavioral scales, facilitates this task in the university context.

## 1. Introduction

The impact of the pandemic has been evident in all areas of society, and the educational setting is no exception. The influence of sanitary restrictions on the educational context has led academia to reflect on the consequences and transformations undergone over the past two years. This fact is evidenced by the numerous studies published on this issue. These publications, among other questions, address changes in teaching methodologies [1], the role of institutional communication systems during the pandemic [2], the students motivation and engagement in the learning processes [3], the pandemic impact on the universities’ international activity [4], or the factors contributing to anxiety disorders in teachers [5].

In this sense, one of the main consequences of the pandemic and the sanitary restrictions that limited face-to-face teaching, is the consolidation of online teaching and blended learning methodologies [6,7]. Consequently, nowadays, the analysis of the mechanisms and instruments used to assess the effectiveness of online teaching and blended learning methodologies has become a topic of crucial importance.

The measurement of teaching effectiveness is a topic that has attracted interest since the early twentieth century, becoming an essential element in educational institutions all around the world [8,9,10,11,12,13,14]. Over the years, a number of studies have examined the instruments used to evaluate teacher effectiveness; nevertheless, these investigations tend to focus on assessing face-to-face instruction [15,16,17,18]. Conversely, studies on the mechanisms used to assess teaching effectiveness in online and blended learning methodologies are very scarce. And furthermore, when this issue is analyzed, the topic is addressed considering instruments that use almost exclusively Likert-type scales (ordinal scales, in which the student expresses his/her degree of agreement with a set of statements related to teacher performance). Some of the most representative works on the analysis of teaching effectiveness in online and blended learning methodologies are those of Bangert [19], García Mestanza [20], and Cañadas and Cuétara [21].

Bangert [19] develops the Student Evaluation of Online Teaching Effectiveness (SEOTE) scale, an instrument with 23 items. This questionnaire shows the existence of four dimensions in online teaching: Student-faculty interaction, Active learning, Task follow-up, and Cooperation among students. The author creates an instrument in which both the fit values of the model (CFI = 0.99; RMSEA = 0.042) and the Cronbach’s alpha statistic (between 0.820 and 0.940) confirm the questionnaire’s robustness.

García Mestanza [20] presents an instrument with 41 items. This questionnaire distinguishes four dimensions in the instruction within virtual environments: Course planning and content, Teacher’s activity, Interaction with the student, and Technical setting. The instrument —explaining 68.44% of the variance— has a poor fit (GFI = 0.872; RMSEA = 0.134; RMSR = 0.061) but an excellent Cronbach’s alpha (0.976).

Along the same lines, Cañadas and Cuétara [21] design a questionnaire with 40 items. The authors reveal the existence of three teaching dimensions in distance instruction methodologies: Teacher’s professionalism, Teacher’s leadership, and Relationship with students. The instrument —explaining 44.67% of the variance— has satisfactory fit values (GFI = 0.96; AGFI = 0.96; RMSEA = 0.078) and again a suitable Cronbach’s alpha (0.928).

### 1.1. Behavioral Anchored Rating Scales (BARS) and the Assessment of Teaching Effectiveness

The evaluation of teaching effectiveness, as indicated above, traditionally focuses on the analysis of face-to-face instruction scenarios and Likert-type questionnaires; however, Behavioral Anchored Rating Scales (BARS) are rarely used for the study of this question.

BARS appeared, in the early 1960s, with the objective of reducing ambiguity when evaluating job performance [22]. Since then, this type of scales have been used to measure effectiveness in very different contexts [23,24,25,26]. In BARS instruments the response options that would be represented on the Likert scale by ordinal positions, indicative of the evaluator’s degree of agreement, are replaced by behavioral episodes representative of the level of effectiveness of the professional who is being evaluated.

In accordance with different authors, part of the success of the BARS lies in its psychometric advantages over other measurement systems such as the Likert-type instruments [27,28,29]. Several investigations demonstrate that BARS tend to produce smaller halo effect and leniency error than other types of scales [30,31]. Other benefits of BARS are improvements in validity and reductions in the influence of bias during the assessment [32,33]. Along the same lines, many studies suggest that behavioral scales provide indicators of better interrater reliability than those found in other questionnaires [32,34].

In summary, the benefits of scales with behavioral episodes are such that even some authors state that BARS are technically, and psychometrically, better than any other measurement instrument [31,35]. This superiority is often attributed to the rigor of scale construction [36], the isolation between the scales that protect them from biases originated in other dimensions [29], the involvement of individuals connected with the activity under evaluation in the instrument construction [37], or even the benefits of using terminology familiar to the rater in the final questionnaire [38].

Within the educational setting, BARS have been used in different stages, programs, and modalities of instruction. Kavanagh and Duffy [39] use this type of questionnaire to evaluate teaching competences at a distance education program. Fernández Millán and Fernández Navas [40] use these scales to evaluate the efficiency of social educators in child protection centers. Hom et al. [41] introduce this system to appraise teachers of a summer school program. Matosas-López et al. [42] postulate the application of scales with behavioral episodes to measure the teaching performance of university professors.

However, despite there are studies on the use of BARS for the assessment of teaching effectiveness and even several studies examining some psychometric attributes [43,44,45], none of these investigations analyze comprehensively the psychometric properties of these questionnaires for the appraisal of teaching effectiveness in online and blended learning methodologies.

### 1.2. Validity and Reliability of the Instruments Used the Assess Teaching Effectiveness

The suitability of the instruments used for the evaluation of teaching effectiveness is one of the issues commonly examined in this field [46,47]. For this task, Kember and Leung [48] indicate that two fundamental criteria must be considered when determining whether an instrument is psychometrically suitable to measure teaching effectiveness: one its validity, the other its reliability.

#### 1.2.1. Validity

Validity can be defined as the degree to which the results obtained with the questionnaire can measure the phenomenon intended to be measured. When examining the validity of a measurement instrument, Spooren et al. [49] indicate the existence of four types of validity: (a) content validity, (b) comprehension validity, (c) construct validity, and (d) confirmation of construct validity.

Content validity concerns the way in which the questionnaire’s items can adequately represent the situation to be assessed. Comprehension validity is the degree of specificity and clarity of the questions in the instrument. Construct validity refers to the extent to which the questionnaire can provide a meaningful assessment of a group of characteristics in different populations. And, finally, the confirmation of construct validity is used to verify the significance of the previous analysis.

The techniques utilized to measure each of these types of validity are obviously different. To measure content validity, the technique commonly used is the expert judgment. In this technique, a panel of judges specialized in the topic evaluates the suitability of each items in the instrument [15,50]. Comprehension validity is generally examined by observing the asymmetry and kurtosis coefficients, as well as the corrected item-total correlation indicators [51,52,53]. Papers that analyze construct validity use exploratory factor analysis (EFA) techniques, measuring the instrument potential based on the percentage of total variance explained [54,55]. And studies that seek to corroborate the robustness of the questionnaire apply confirmatory factor analysis (CFA), monitoring indicators such as the comparative fit index (CFI), goodness of fit index (GFI), adjusted goodness of fit index (AGFI), root mean square error of approximation (RMSEA), or standardized root mean square error residuals (SRMR), among others [48,56].

The EFA and CFA techniques allow the researcher to understand the structure of constructs underlying the instrument and, consequently, the teaching dimensions to be considered. Although some authors defend the possibility of considering a single dimension as an overall measurement of teaching effectiveness [57,58], the majority of the researches believe that the wide number of aspects intrinsic to the teaching activity requires the concept to be addressed by a multidimensional approach [56,59].

#### 1.2.2. Reliability

Likewise, reliability refers to the level of consistency observed in the responses of the evaluators to the different items of the instrument for each subject assessed. While Sun et al. [60], for example, postulate the use of generalizability theory, or G theory, as a statistical framework for analyzing the reliability of these instruments, the most widespread technique for examining reliability employs Cronbach’s alpha coefficient as indicator.

This coefficient explores the homogeneity of the items in the questionnaire, revealing whether they are interconnected with each other in the instrument’s factor structure [61]. This yields a measurement of the precision with which the set of items considered can measure the phenomenon under study.

Although the Cronbach’s alpha statistic is widely accepted as an indicator of reliability, Kember and Leung [48] emphasize that this coefficient is conditioned by two aspects: the length of the questionnaire and the number of dimensions considered.

Cronbach’s alpha tends to increase as more questions are added to the instrument but increasing the length of the questionnaire may discourage participation in the survey. Similarly, Cronbach’s alpha increases in instruments with one or a few dimensions. Nonetheless, as indicated above, given the complex nature of teaching, this activity is typically addressed using multidimensional approaches. These two aspects, according to Kember and Leung [48], compel researchers to navigate the dilemma between increasing the instrument reliability or using the appropriate number of items and dimensions.

According to some authors [53,62], other indicators that can be used to verify the reliability of these questionnaire are the average variance extracted (AVE) and the composite reliability. In both cases, these indicators are used to analyze the internal consistency of the instrument as a whole.

### 1.3. Objective

While it is true that the literature on online and blended learning methodologies includes psychometric analysis on instruments that use Likert-type questionnaires (such as those mentioned above) the same is not true for studies that explore the validity and reliability of instruments that use BARS [63].

The literature review conducted, exploring—in line with authors such as Spooren et al. [64], Uttl et al. [65] or Moreno Guerrero [66]—the Web of Science and Scopus catalogues, reveals the lack of specific publications on the validity and reliability of BARS for the measurement of teaching effectiveness in online or blended learning settings. In this sense, although certain publications on this topic can be identified in other minor databases such as ERIC or SCIELO, these publications are limited, both in number and scope, reinforcing the researchers position in the need for studies of this nature.

The present paper aims to examine the validity and reliability of a BARS-type instrument designed to assess the effectiveness of university professors who teach in blended learning modalities. Accordingly, the authors pose the following research questions:

RQ1: Is the BARS questionnaire examined a valid instrument to assess teaching effectiveness in blended learning methodologies?
RQ2: Is the BARS questionnaire examined a reliable instrument to assess teaching effectiveness in blended learning methodologies?

In light of the above, this research contributes to fill an important gap in the literature; analyzing the validity and reliability of an instrument with behavioral scales intended to appraise teaching effectiveness in blended learning modalities in the university setting.

## 2. Materials and Methods

### 2.1. Instrument

The BARS instrument under analysis was designed, in Spain, by researchers from a medium size university, to measure the effectiveness of university professors in blended learning environments [67]. The questionnaire was designed with the participation of 477 students, together with a panel of six professors at the same university who were experts in this teaching modality. In line with previous research on the design of BARS, the instrument was constructed through several stages of refinement, based on behavioral episodes representative of teacher performance gathered with the involvement of the students and teachers already mentioned [33,68].

The instrument construction consisted of six stages: (a) the teaching categories of the blended learning methodology were defined by the panel of teachers; (b) for each category, behavioral examples of effective, and ineffective, teaching were collected with the participation of the students using unstructured interviews; (c) behavioral episodes were filtered to eliminate duplicate or ambiguous episodes; (d) the behavioral examples were reclassified into the teaching categories initially considered; (e) behavioral episodes were clustered into groups of core behavioral aspects; (f) and finally, behavioral examples were selected to illustrate the anchor points, representative of each level of effectiveness, in each category of the blended learning methodology considered in the questionnaire.

The final instrument contained ten questions, or items, to assess ten categories of instruction in blended learning environments. The categories in the questionnaire were: Course introduction, Evaluation system description, Time management, Organizational consistency, Evaluation system implementation, Dealing with doubts, Explicative capacity, General availability, Follow-up easiness, and General satisfaction. The instrument—whose psychometric suitability is the object of analysis in this paper—is presented entirely in the Appendix A.

### 2.2. Participants

The research involved a sample of 1436 students out of the 39,892 enrolled in the same Spanish university in which the instrument was designed. All the participants involved in the study were undergraduate students, with previous experiences in online or blended learning modalities. The researchers selected the sample participants by convenience sampling. Establishing a confidence level of 98%, the researchers worked with a sampling error of 3.02%. Since it is common to accept sampling errors of up to 5% [69], the margin of error considered ensures that the sample has an appropriate statistical significance.

The participants were enrolled in different programs and courses within the area of social sciences studies: Marketing (19.50%), Business Administration (17.64%), Education (15.72%), Journalism (13.31%), International Relations (12.58%), Political Sciences (11.09%), and Law (10.16%). The average age of the participants was 21.48 years (with a standard deviation of 3.07), being 54.70% of them women, and 45.30% men.

During the study, 117 educators were evaluated out of a total of 380 teachers from the studies in social sciences. With a confidence level of 80%, the researchers worked with a sampling error of 4.90%, values again accepted in the context of educational research [69].

The substantial sample of research participants (students, on the one hand, and teachers, on the other) required the development of the investigation over several successive years. So, the research was carried out between 2019 and 2022, covering the academic years 2019–20, 2020–21 and 2021–22.

### 2.3. Phases of the Analysis

Many of the psychometric studies of instruments that evaluate teaching effectiveness begin with an analysis of content validity. However, in authors’ opinion, the thoroughness and accuracy required to construct BARS offer sufficient guarantees to omit that step. The direct involvement of students and teachers in the design of BARS, besides the use of behavioral episodes to represent the anchor points on the scale, ensures the suitability of the content for the questionnaire purposes.

In line with previous instrumental studies in the university context [70,71], the authors carry out a psychometric study of four phases: (1) comprehension validity analysis, (2) construct validity analysis, (3) confirmation of construct validity, and (4) analysis of the instrument reliability. In addition, the paper presents, also, the descriptive results obtained with the questionnaire examined.

According to previous research [51,52,53], in order to analyze comprehension validity, the authors examine the asymmetry and kurtosis coefficients, as well as the corrected item-total correlation indicators.

Construct validity, in line with previous studies [72,73,74], is addressed using EFA followed by CFA. The researchers, following the recommendations of previous studies [16,75], examine the indicators CFI, GFI, AGFI, RMSEA, and SRMR.

Finally, the reliability analysis is carried out considering the Cronbach’s alpha coefficient [21,48,76], as well as the AVE and the composite reliability [77].

After the validity and reliability study, the authors present, also, the descriptive results obtained whit the instrument providing the mean and standard deviation for each question. All the analyses were performed in the IBM SPSS 27 statistical analysis package, as well its extension AMOS 20 for confirmatory tests.

## 3. Results

### 3.1. Comprehension Validity Analysis

In comprehension validity analysis, items with asymmetry and kurtosis values between −1 and 1 are considered adequate [52]. Accordingly, as it is shown in Table 1, the asymmetry and kurtosis coefficients are optimal for the ten items of the instrument.

Similarly, the discrimination level of each item is examined by observing the corrected item-total indicators. In accordance with Lacave Rodero et al. [51], items with corrected item-total values above 0.20 are considered adequate. Table 1 indicates that correlation values are acceptable for all ten questions.

The asymmetry and kurtosis coefficients, as well as the corrected item-total correlation indicators, recommend keeping all the items in the instrument. The questionnaire thus shows suitable comprehension validity.

### 3.2. Construct Validity Analysis

Before performing the EFA required to examine construct validity, the Kaiser-Meyer-Olkin test for sampling adequacy and Bartlett’s test of sphericity are calculated to assess the relevance of the analysis. The Kaiser-Meyer-Olkin value is 0.939, exceeding the recommended value of 0.600. Bartlett’s test of sphericity reaches a significance of 0.000. Both results reveal the existence of sufficient correlations between the instrument’s items, thus corroborating the appropriateness of the EFA.

**Figure 1:**
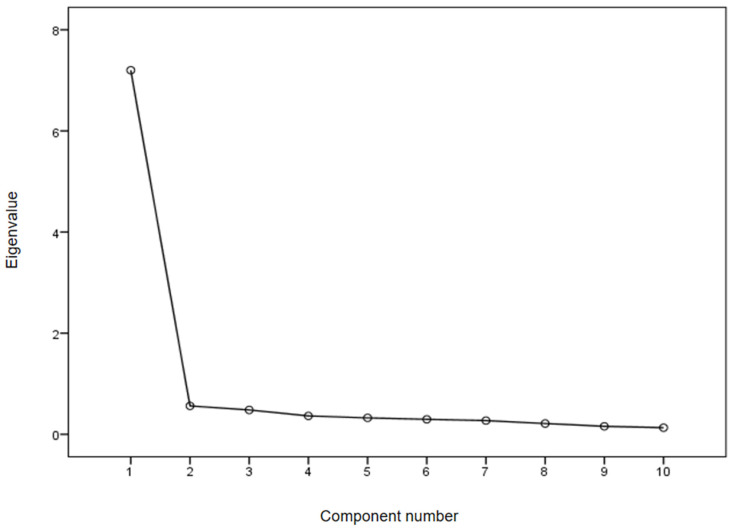
Dimensions in the scree plot.

As preliminary approach, the authors examine the scree plot (Figure 1), hence anticipating the existence of two, clearly differentiated, factors or dimensions.

The EFA is performed following the principal component extraction method, with Varimax rotation, applying the criterion of eigenvalues greater than 1 for the factor extraction. The rotated component matrix extracted shows the dimensional structure of the instrument (see Table 2), confirming the existence of two underlying factors, as indicated in Figure 1.

These two factors explain 77.61% of the instrument’s total variance. The details of the composition of each of the constructs are described below.

- Factor 1. The construct with six items (General satisfaction, Follow-up easiness, Dealing with doubts, General availability, Explicative capacity, and Time management) explains 42.92% of the variance. This construct encompasses aspects related to the teacher’s skills (for example, dealing with doubts or explicative capacity), as well as others that refer to the teacher’s attitude during the course (for example, follow-up ease or availability). This factor is named by the authors as Teacher’s Aptitude and Attitude.
- Factor 2. The construct with four items (Evaluation system implementation, Course introduction, Evaluation system description, and Organizational consistency) explains 34.68% of the variance. This construct involves aspects pertaining to the presentation and organization of the course, as well as those related to the evaluation system. The researchers name this factor Structure and Evaluation.

### 3.3. Confirmation of Construct Validity

**Figure 2:**
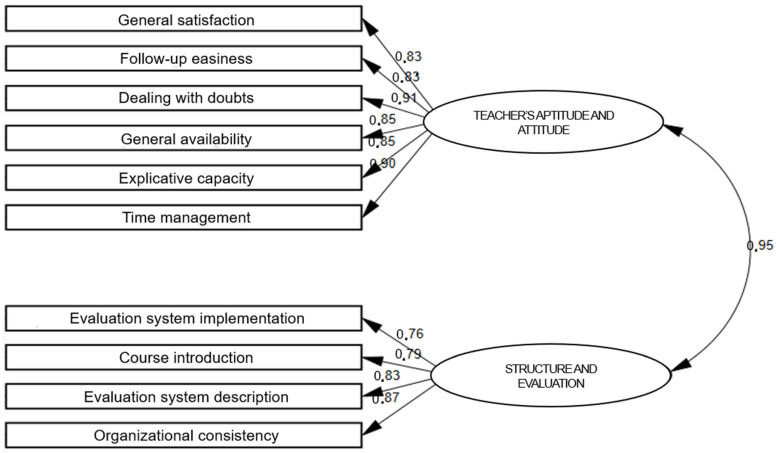
Dimensional model derived from the CFA.

Once the dimensional structure of the instrument is known, its validity is confirmed using a CFA. This analysis makes it possible to corroborate the extent to which the data support the factor structure initially found during the EFA. The CFA is performed by estimating the parameters of the model based on the maximum likelihood criterion. The model and the associations between the ten items are presented in Figure 2, which also shows the standardized regression coefficients.

The authors, in line with Martínez Clares et al. [78], supplement the information in Figure 2 by presenting the estimation parameter, standard error (SE), and critical ratio (CR) of both the associations between items and factors, as well as the correspondences between the two factors identified in the instrument (see Table 3). The data show satisfactory regression coefficients, ranging from 0.906 (Dealing with doubts <=> Teacher’s Aptitude and Attitude) to 0.759 (Evaluation system implementation <=> Structure and Evaluation).

To conclude this part of the analysis, the following indicators are examined: CFI, GFI, AGFI, RMSEA, SRMR [16,75], and, as another measure of model fit, the chi-squared ratio over degrees of freedom (Table 4).

The CFI, as a comparative fit coefficient, is considered adequate with a value above 0.90 [79]. The GFI and AGFI, both representative of the combined degree of fit, are also optimal at values above 0.90 [80]. The RMSEA, as an estimator of the model’s residual value, indicates a sufficient fit at a value lower than 0.05 [81]. Finally, the SRMR, as an indicator of the status of the standardized residuals, suggests an optimal fit at a value below 0.08 [82].

### 3.4. Analysis of the Instrument Reliability

The questionnaire’s reliability, as a whole, presents a Cronbach’s alpha coefficient of 0.956. The observation of the coefficients for each of the two dimensions identified also corroborates the internal consistency of the items comprising each factor. The first construct has a Cronbach’s alpha coefficient of 0.945, while the second has a Cronbach’s alpha of 0.886. In line with George and Mallery [83], the indicators for the instrument as a whole besides the coefficient for the first dimension, both above 0.900, can be considered excellent. While the coefficient for the second dimension, which is between 0.800 and 0.900, can be considered good.

In addition to the Cronbach’s alpha statistic, the authors, in line with Martín García et al. [77], also examine the average variance extracted (AVE) and the composite reliability. These two indicators are above the recommended values of 0.500 for the former [53] and 0.700 for the latter [62], corroborating once again the instrument reliability (see Table 5).

### 3.5. Descriptive Results Obtained with the Instrument

Finally, Table 6 presents the descriptive results obtained with the BARS for each item, grouped according to the two dimensions identified during the factor analyses. In the Teacher’s Aptitude and Attitude dimension, the results obtained for the teachers evaluated during the investigation are particularly noteworthy regarding follow-up easiness and time management.

The Structure and Evaluation dimension contains scores that are generally high and have lower levels of dispersion than the ones for the first dimension. The scores obtained by the teachers regarding the application of the evaluation system and the course organization are also remarkable.

## 4. Discussion and Conclusions

In accordance with different authors, teaching effectiveness can be defined as the efficacy and productivity of the lecturers in the performance of their duties [84,85,86]. Even though it is true that instruments used to measure teaching effectiveness adopt different formats [87,88], even taking qualitative approaches [89], in most cases they are presented in the form of Likert scales. Studies such as those of Muñoz-Cantero et al. [90], González-López and López-Cámara [91], Lizasoain-Hernández et al. [16], or Leguey-Galan et al. [92], among others, corroborate that almost all universities use Likert questionnaires for this purpose. Despite this, there are also authors, and studies, that use BARS to measure teaching effectiveness [44,67]. Here stand up investigations comparing BARS-type instruments with other sorts of questionnaires [45,93], or research on the practical application and distribution of these surveys [33,94]; however, there are few comprehensive studies on the validity and reliability of BARS questionnaires.

At a time in which the sanitary restrictions have definitively consolidate blended learning methodologies, the results obtained provide a positive answer to the research questions posed by the authors, indicating that the BARS instrument examined can be perfectly valid (RQ1) and reliable (RQ2) for the evaluation of teaching effectiveness in this setting. The findings of this research complement the study carried out by Matosas-López et al. [67], demonstrating that the questionnaire proposed by these authors is solid and consistent in psychometric terms. This conclusion is justified by the results obtained in the four phases of the current psychometric study.

The comprehension validity analysis (RQ1) presents optimal asymmetry, kurtosis, and corrected item-total correlation values for all the items in the questionnaire. The construct validity, explored through EFA, reveals the existence of two dimensions (Teacher’s Aptitude and Attitude and Structure and Evaluation) that can explain 77.61% of the variance in teaching effectiveness in blended learning methodologies. These data show an explanatory power far higher than that offered by other instruments with Likert scales that are designed to assess teaching in distance modalities, such as those of García Mestanza [20] or Cañadas and Cuétara [21]. Those studies present questionnaires that explain 68.44% and 44.67% of the variance, respectively, the former with a four-dimensional instrument and the latter with a three-dimensional questionnaire. Although the BARS instrument examined here considers fewer dimensions, it presents a higher explanatory power. This aspect is particularly significant if we consider the transformations recently experienced in the context of distance teaching.

The CFA, performed to corroborate the construct validity (RQ1), shows CFI, GFI, AGFI, RMSEA, and SRMR values within the usual thresholds. The comparison between these fit values and those presented for previous Likert-type questionnaires reveals the robustness of the proposed instrument. For example, the RMSEA of 0.042 reflects a better fit than that presented by García Mestanza [20] (RMSEA = 0.134) or Cañadas and Cuétara [21] (RMSEA = 0.078) and an identical fit to that shown by Bangert [19] in his SEOTE questionnaire.

The findings also reveal how the instrument remains aligned with the multidimensional approach of these measurements, but without using too many dimensions. The dimensions detected—Teacher’s Aptitude and Attitude and Structure and Evaluation—are also in line with the dimensions identified in previous psychometric studies that have used Likert-type questionnaires to measure teaching effectiveness in distance learning modalities. The aspects related to the teacher’s aptitude and attitude can be observed in the Teacher’s activity dimension in García Mestanza [20], as well as in the Teacher’s professionalism and Teacher’s leadership dimensions in Cañadas and Cuétara [21]. Similarly, the aspects related to the structure and evaluation of the course are observed in the Course planning and content and Technical environment dimensions in García Mestanza [20]. Correspondingly, the identification of a small number of dimensions, according to Kember and Leung [48], results in a more reliable instrument, as it is reflected by the Cronbach’s alpha obtained.

Furthermore, the two dimensions detected (Teacher’s Aptitude and Attitude and Structure and Evaluation), in line with similar studies, show the importance of both the teacher’s skills [95,96] and course design and organization in these scenarios of distance instruction [97,98]. Being aware of both issues is critically important at a time in which online and blended learning practices are definitively established on higher education institutions [99,100].

Finally, the reliability analysis (RQ2) reveals a high Cronbach’s alpha coefficient of 0.956 for the instrument as a whole. This value exceeds the reliability values obtained for the Likert-type questionnaires of Bangert [19] and Cañadas and Cuétara [21]; the first with Cronbach’s alpha values between 0.820 and 0.940 and the second with a coefficient of 0.928.

The findings obtained corroborate the instrument’s optimal validity (RQ1) and reliability (RQ2). This fact confirms the approach of other authors [29,101] who had already emphasized the potential of BARS to provide higher levels of validity and reliability than other types of questionnaires. Not only due to the development of mutually independent scales for each performance category, but also thanks to the use of behavioral examples in the representation of each anchor point on the scale.

Given the above, the authors conclude that this paper fills a gap in the literature by presenting a robust instrument for measuring teaching effectiveness in blended learning environments in the university setting.

### Limitations and Directions for Future Research

Although, in the authors’ opinion, the paper contributes significantly to the literature on this field, the investigation also has several limitations. The limitations are primarily related to the sample of participants. Although the sample is significant given the population under study, this issue could be improved in two ways. First, the sample included only students from the area of social sciences programs. Second, the research was carried out exclusively at a Spanish university.

Even though, in the present study no differences were observed in the results between the different fields analyzed, and there are patterns in the assessment of teaching effectiveness that transcend disciplines or geographical locations, it may be interesting to conduct psychometric studies on samples from the fields of health sciences, engineering, or humanities, and it may also be valuable to recreate this analysis in different countries.

Finally, other limitations in the study are the lack of exploration of the potential pedagogical implications of these evaluations and the absence of the analysis of the sociodemographic bias (gender, sex, years of study, etc.) in the success of these instruments.

These lines of research would contribute to understanding the degree to which the good validity and reliability results obtained in this study can be extrapolated—or not—to other populations. This would reveal the extent to which the instrument (presented in the Appendix A) could be used in different universities around the world to evaluate teaching effectiveness in blended learning methodologies in the university setting.

## Figures and Tables

**Table 1:** Asymmetry and kurtosis coefficients and corrected item-total correlation indicators.

Item	Asymmetry	Kurtosis	Corrected Item-Total Correction
Course introduction	−0.441	−0.424	0.754
Evaluation system description	−0.245	−0.232	0.793
Time management	−0.062	−0.730	0.874
General availability	0.187	−0.433	0.833
Organizational consistency	0.054	−0.627	0.830
Evaluation system implementation	−0.145	−0.465	0.718
Dealing with doubts	0.236	−0.594	0.874
Explicative capacity	0.435	−0.276	0.830
Follow-up easiness	0.161	−0.559	0.791
General satisfaction	0.393	−0.571	0.797

Source: The authors.

**Table 2:** Rotated component matrix.

Item	Factor 1	Factor 2
General satisfaction	0.828	
Follow-up easiness	0.799	
Dealing with doubts	0.785	
General availability	0.737	
Explicative capacity	0.736	
Time management	0.701	
Evaluation system implementation		0.783
Course introduction		0.775
Evaluation system description		0.769
Organizational consistency		0.659

Source: The authors.

**Table 3:** Regression coefficients and standardized regression coefficients between items and factors.

			Regression Coefficients			Standardized Coefficients
Relationship between Items and Factors			Estimation	SE	CR	Estimation
General satisfaction	<=>	Teacher’s Aptitude and Attitude	0.908	0.04	23.82	0.831
Follow-up easiness	<=>	Teacher’s Aptitude and Attitude	0.863	0.04	23.62	0.827
Dealing with doubts	<=>	Teacher’s Aptitude and Attitude	0.983	0.03	29.05	0.906
General availability	<=>	Teacher’s Aptitude and Attitude	0.858	0.03	25.02	0.850
Explicative capacity	<=>	Teacher’s Aptitude and Attitude	0.867	0.04	25.06	0.851
Time management	<=>	Teacher’s Aptitude and Attitude	1			0.899
Evaluation system implementation	<=>	Structure and Evaluation	0.786	0.04	18.79	0.759
Course introduction	<=>	Structure and Evaluation	0.879	0.04	2.22	0.795
Evaluation system description	<=>	Structure and Evaluation	0.825	0.04	21.93	0.833
Organizational consistency	<=>	Structure and Evaluation	1			0.866
Teacher’s Aptitude and Attitude	<=>	Structure and Evaluation	1.999	0.16	12.27	0.947

Source: The authors.

**Table 4:** CFA fit statistics.

Indicator	Usual Threshold	Value Obtained
χ^2^/g.l. (Chi-squared ratio/Degrees of freedom)	<3.00	2.091
CFI (Comparative fit index)	>0.90	0.940
GFI (Goodness of fit index)	>0.90	0.920
AGFI (Adjusted goodness of fit index)	>0.90	0.902
RMSEA (Root mean square error of approximation)	<0.05	0.042
SRMR (Standardized root mean square error residuals)	<0.08	0.027

Source: The authors.

**Table 5:** Internal consistency indicators.

Factor	Cronbach’s Alpha	AVE	Composite Reliability
Teacher’s Aptitude and Attitude	0.945	0.586	0.894
Structure and Evaluation	0.886	0.560	0.835

Source: The authors.

**Table 6:** Descriptive results obtained with the instrument.

Item	Mean (Values from 1 to 5)	SD
Teacher’s Aptitude and Attitude		
General satisfaction	3.01	1.279
Follow-up easiness	3.11	1.395
Dealing with doubts	2.75	1.248
General availability	2.98	1.173
Explicative capacity	2.79	1.279
Time management	3.14	1.189
Structure and Evaluation		
Evaluation system implementation	3.53	1.022
Course introduction	3.48	1.089
Evaluation system description	3.39	1.072
Organizational consistency	3.55	1.101

Source: The authors.

## Data Availability

Not applicable.
